# A Spider-Joint-like Bionic Actuator with an Approximately Triangular Prism Shape

**DOI:** 10.3390/biomimetics8030299

**Published:** 2023-07-09

**Authors:** Xiaomao Jiang, Jun Yang, Le Zeng, Changyang Huang

**Affiliations:** 1 College of Engineering and Design, Hunan Normal University, Changsha 410081, China; 17873941901@163.com (X.J.); hcydwyy@163.com (C.H.); 2 Department of Aviation Machinery Manufacturing, Changsha Aeronautical Vocational and Technical College, Changsha 410124, China; zenglewish@163.com

**Keywords:** soft robot, actuator, bionic, spider inspired, fluidic

## Abstract

The unique drive principle and strong manipulation ability of spider legs have led to several bionic robot designs. However, some parameters of bionic actuators still need to be improved, such as torque. Inspired by the hydraulic drive principle of spider legs, this paper describes the design of a bionic actuator characterized by the use of air pressure on each surface and its transmittance in the direction of movement, achieving a torque amplification effect. The produced torque is as high as 4.78 N m. In addition, its torque characteristics during folding motions are similar to those during unfolding motions, showing that the bionic actuator has stable bidirectional drive capability.

## 1. Introduction

Soft robots have good adaptability and can be embedded into robotic structures [1,2,3,4]. The versatility of natural creatures can be reproduced in soft robots by incorporating soft elements into their robotic structure. Unlike traditional robots, soft robots can effectively interact with an uncertain external environment, have large-scale continuous deformation abilities, and are strongly adaptable to their environment [5]. The soft actuator is the key component of the soft robot and plays a key role in its performance. The design inspiration for soft actuators usually comes from natural creatures [6,7,8]. Bionic soft actuators often resemble natural creatures in terms of function, structure, and mode of movement, such as in the example of an octopus-inspired actuator, among others [9,10,11,12]. When designing soft actuators, the design of the constraint structures is particularly important. The constraint structure is the key factor affecting the performance of the soft actuator [13,14,15]. Regular deformation of the actuator can be achieved using constraints [16]. The drive function is realized by rationally using the deformation of the actuator. However, these constraints impede the movement of the actuator, resulting in its poor performance. In addition, once filled with compressed air, the soft actuator may show some inherent defects, such as inaccurate movement and a fragile wall, but this is unavoidable in practical applications [17,18,19,20]. 

**Figure 1:**
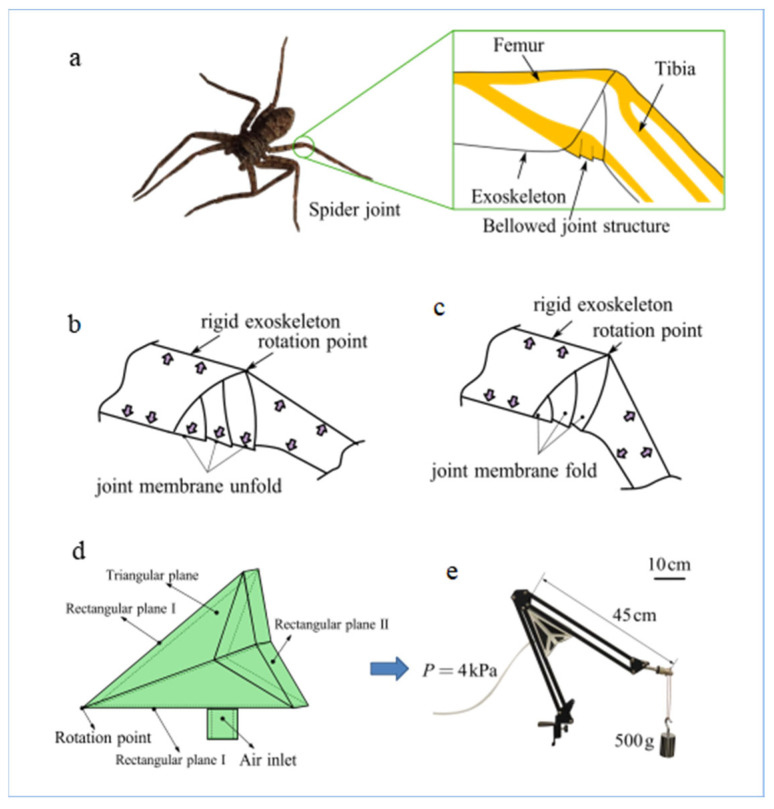
(**a**) Spider joint shape for joint membrane folding.(**b**) The joint membrane unfolds when the spider’s legs extend. (**c**) The joint membrane folds when the spider’s legs bend. (**d**) Bioinspired actuator. (**e**) The bionic actuator allows the mechanical arm to lift a 500 g hammer. The torque of gravity during the ascent of the mechanical arm and the torque of the bionic actuator increase as the angle increases. The mechanical arm can rise stably with a constant pressure of 4 kpa.

Adopting a folding structure offers a solution to the above problems. Applying a folding structure to a soft actuator can protect it and produce accurate movement. Applying a folding structure to a soft actuator increases its rigidity and anisotropy and reduces its weight [21,22,23]. Efficient folding structures are sometimes inspired by arthropods. Spiders extend their legs using hydraulic pressure instead of muscle pairs, providing a good model for actuator designs. This unique form of drive allows the spider’s legs to perform precise and coordinated movements [24,25,26]. Spider legs comprise a rigid exoskeleton and flexible joints; the rigid exoskeleton is divided by flexible joints, with a soft and collapsible joint membrane on the ventral side of the joint [27,28,29]. Each joint membrane expands and squeezes another to trigger joint rotation. The joint membrane folds when the spider’s legs bend. The use of a joint membrane in the structure allows the entire spider leg to form a closed cavity, as shown in Figure 1a-c. An increase in hemolymph pressure at the femur–tibia joints of the spider’s legs is required for the joint membranes to expand and thus squeeze each other to stimulate joint rotation. An approximately triangular prism-shaped foldable structure was obtained based on imitating a spider’s leg joint, as shown in Figure 1d. The specific folding scheme is as follows: The approximately triangular prismatic main body consists of two triangular planes, two rectangular planes (I), and one rectangular plane (II). Rectangular plane I is based on the bionics of an exoskeleton, and the triangular plane and rectangular plane II are based on the bionics of a joint membrane and play a role in sealing the structure. Furthermore, the design for a bionic actuator according to this structure and its application to lifting equipment are shown in Figure 1e.

This paper is organized as follows: In Section 1, the research status of soft robot actuators is introduced, and the structure of spider leg joints is studied, and a spider-joint-like bionic actuator with an approximately triangular prism shape is proposed. In Section 2, the structure of the actuator is designed and elaborated upon, the motion characteristics and torque characteristics of the actuator are analyzed, and the production method of the bionic actuator is introduced. In Section 3, experiments are carried out on the bionic actuator, and the output torque values of the actuator under different angles and pressures are measured. The torque characteristics of forward and reverse driving are compared, and the bidirectional motion capability of the actuator is expounded. Moreover, by combining theory and experiment, the bistable characteristics of the spider-joint-like bionic actuator with an approximately triangular prism shape are verified. In Section 4, the influence of the position of point N on the bionic actuator on the performance of the drive is discussed, and the parameters of the actuator are optimized. In Section 5, the work carried out in this paper is summarized.

## 2. Design and Production

### 2.1. Structural Design

**Figure 2:**
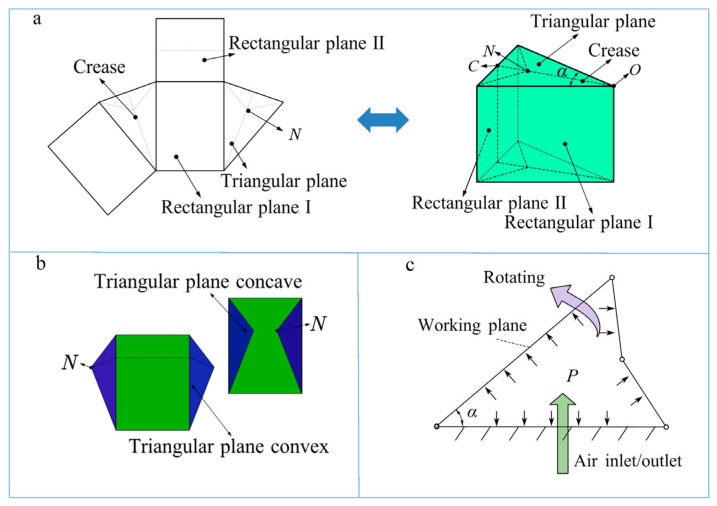
(**a**) The unfolding form of the structure: the dotted line is a crease, and all the edges and creases are soft. (**b**) The approximate triangular prism origami structure to be folded, wherein the triangular plane may be concave or convex. (**c**) *α* is the angle between two working planes, and *P* is the input air pressure. When the bionic actuator is inflated, the two working planes unfold, and *α* increases.

The folding scheme is as follows: The approximately triangular prismatic main body consists of two triangular planes, two rectangular planes (I), and one rectangular plane (II), and all planes form a closed structure, as shown in Figure 2a. There are creases in rectangular plane II, each triangular plane has three creases along its bisector, and the three creases intersect at point N. Suppose each plane is a rigid plane, with the planes connected at their edges, and all creases and edges are soft. The structure can be folded along the edges, and creases and can be folded into a plane, as shown in Figure 2b. The position of point N determines whether the triangular plane is concave or convex.

When the actuator is filled with air, the angle (*α*) between the two rectangular planes (I) increases, and the entire structure unfolds until it reaches the maximum. The maximum of *α* is determined by the dimensions of each edge. During unfolding, air pressure acts on all interior surfaces of the bionic actuator. The unfolding direction of rectangular plane I is the direction of movement. Rectangular plane I is the working plane, and the pressure of the rectangular plane I directly drives movement. The pressure of rectangular plane II and the triangular plane is also transmitted in the direction of movement through the connecting edge, thereby promoting the unfolding movement of rectangular plane I, as shown in Figure 2c.

The structure folds when the internal pressure of the bionic actuator is lower than the atmospheric pressure.

### 2.2. Structural Analysis

#### 2.2.1. Output Torque Characteristics of the Bionic Actuators

**Figure 3:**
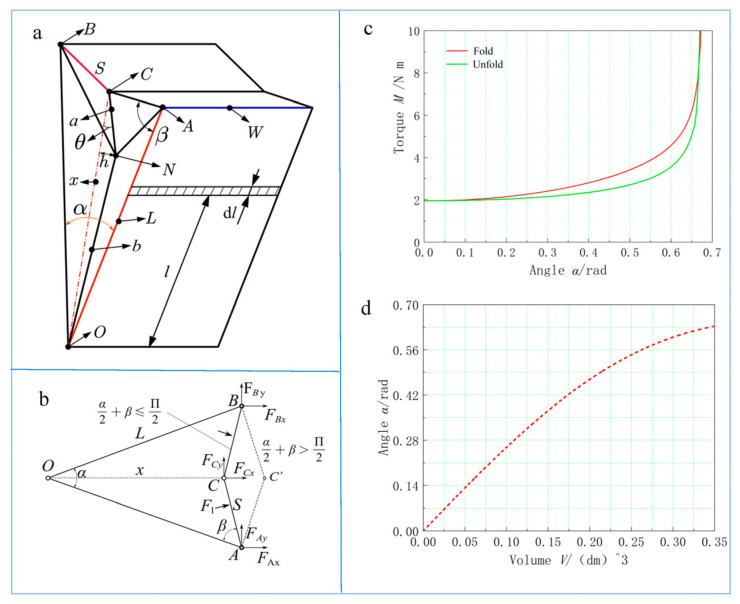
(**a**) The structure of the partially folded bionic actuator. At this time, the triangular plane and rectangular plane II are partially folded, and the triangular plane changes into a quadrangular pyramid shape. (**b**) The force of the bionic actuator is analyzed using a simplified mechanical model. The relationship between (**c**) torque (*M*) and angle (*α*) and (**d**) angle (*α*) and volume (*V*) after calculation and analysis.

To simplify the model, the crease on the triangular plane and rectangular plane II intersects at point *C*. ∠CAO=∠*β*, ∠NCO=∠θ, *a* is the length of *CN*, *x* is the length of *CO*, *b* is the length of *NO*, and *h* is the distance from point *N* to the triangular plane. Because of the structural constraint, rectangular plane II can only be concave, as shown in Figure 3a. The relative pressure of the input air is *p*, the force on rectangular plane I is *F*_1_, the torque generated by the pressure on rectangular plane I is *M*_1_, and the force acting on rectangular plane II produces torque (*M*_2_) through the edge to rectangular plane I. The total torque is *M*; then,

(1) $$M_{1}=\int_{0}^{L}\mathrm{PWl}\mathrm{dl}$$

Based on this structure, we can analyze the stress situation, as shown in Figure 3b.

(2) $$\frac{S}{\sin\frac{\alpha }{2}}=\frac{L}{\sin\left[\pi -\left(\frac{\alpha }{2}+\beta \right)\right]}$$

(3) $$\beta =\sin^{-1}\left(\frac{L}{S}\sin\frac{\alpha }{2}\right)-\frac{\alpha }{2}$$

Then,

(4) $$\{\begin{array}{c} F_{1}=PSW\\ F_{Ax}+F_{Bx}-2F_{1}\sin\left(\frac{\alpha }{2}+\beta \right)=0\\ F_{Ax}=F_{Bx}\;\\ F_{Ay}-F_{By}=0\\ F_{Ax}+F_{Cx}-F_{1}\sin\left(\frac{\alpha }{2}+\beta \right)=0\;\\ F_{Ay}-F_{Cy}+F_{1}\cos\left(\frac{\alpha }{2}+\beta \right)=0\\ F_{Cy}\times S\times \cos\left(\frac{\alpha }{2}+\beta \right)-\frac{1}{2}F_{1}S=0\\ M_{2}=-F_{Ax}\times L\times \sin\frac{\alpha }{2}+F_{Ay}\times L\times \cos\frac{\alpha }{2} \end{array}$$

When the actuator unfolds, the torque is

(5) $$M_{2}=\frac{-F_{1}L^{2}\times \sin^{2}\frac{\alpha }{2}}{S}+\left(\frac{F_{1}}{2\sqrt{1-\frac{L^{2}}{S^{2}}\sin^{2}\frac{\alpha }{2}}}-F_{1}\sqrt{1-\frac{L^{2}}{S^{2}}\sin^{2}\frac{\alpha }{2}}\right)\times L\times \cos\frac{\alpha }{2}$$

When the actuator folds, the torque is

(6) $$M_{2}=\frac{F_{1}L^{2}\times \sin^{2}\frac{\alpha }{2}}{S}-\left(F_{1}\sqrt{1-\frac{L^{2}}{S^{2}}\sin^{2}\frac{\alpha }{2}}-\frac{F_{1}}{2\sqrt{1-\frac{L^{2}}{S^{2}}\sin^{2}\frac{\alpha }{2}}}\right)\times L\times \cos\frac{\alpha }{2}$$

Ignoring the driving effect of air pressure acting on the triangular plane, the total torque is

(7) $$M=M_{1}+M_{2}$$

The following values are set: *P* = 4 kPa, L=0.135 m,W=0.08 m, S=0.045 m, a=0.029 m, and b=0.085 m. The relationship between the total output torque, *M*, and the angle, *α*, is shown in Figure 3c. When the actuator unfolds, the torque of the bionic actuator remains constant at small angles (*α*), and at large angles (*α*), the output torque increases with the increasing angle (*α*); the reinforcing effect of rectangular plane II on the torque increases with the increasing angle (*α*). When the actuator folds, its torque is similar to that of the actuator when it unfolds, but the torque ramps up faster.

#### 2.2.2. The Relationship between the Volume of the Bionic Actuator and the Angle (α)

Ignoring the wall thickness of the bionic actuator, the volume is *V*:

(8) $$V=\frac{1}{2}WL^{2}\sin\alpha -\frac{2}{3}Lh\sin\frac{\alpha }{2}\left(L\times \cos\frac{\alpha }{2}-S\sqrt{1-\frac{L^{2}}{S^{2}}\sin^{2}\frac{\alpha }{2}}\right)-WLS\sin\frac{\alpha }{2}\sqrt{1-\frac{L^{2}}{S^{2}}\sin^{2}\frac{\alpha }{2}}$$

(9) $$h=a\sin\theta =\frac{\sqrt{{\left(2ax\right)}^{2}-{\left(a^{2}+x^{2}-b^{2}\right)}^{2}}}{2x}$$

(10) $$x=L\cos\frac{\alpha }{2}-S\sqrt{1-\frac{L^{2}}{S^{2}}\sin^{2}\frac{\alpha }{2}}$$

Namely,

(11) $$V=WL^{2}\sin\frac{\alpha }{2}\left(\cos\frac{\alpha }{2}-\sqrt{\frac{S^{2}}{L^{2}}-\sin^{2}\frac{\alpha }{2}}\right)-\frac{1}{3}L\sin\frac{\alpha }{2}\sqrt{{\left(2ax\right)}^{2}-{\left(a^{2}+x^{2}-b^{2}\right)}^{2}}$$

The relationship between the angle, *α*, and the volume, V, is shown in Figure 3d. It shows that if the same volume of air fills the space for a given time, *α* increases, but the rate of change in *α* decreases; the reason is that the air pushes the triangular plane and rectangular plane II, moving them outward while causing rectangular plane Ⅰ to unfold. Therefore, as the angle increases, there is a decrease in the movement speed of the actuator.

### 2.3. Bionic Actuator Production Program

**Figure 4:**
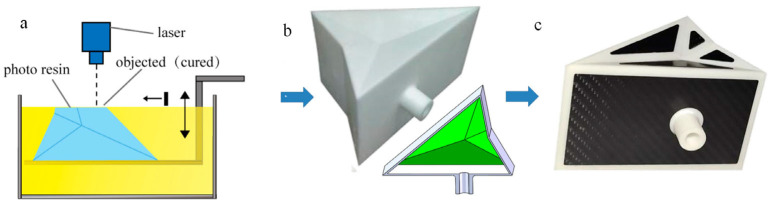
(**a**) The working principle of DLP printing technology. (**b**) The soft actuator body processed using 3D printing. (**c**) The soft actuator body structure and rigid carbon fiber plate are bonded using an adhesive to form a rigid and soft bionic actuator.

In the above structure, all planes are rigid, and all edges and creases are soft. The manufacturing steps are as follows: 

Step 1: The actuator body is produced by applying 3D printing technology, as shown in Figure 4a,b.

The actuator needs to have good air tightness to meet the requirements, and each side and crease must have enough softness. We chose soft rubber resin as the raw material for 3D printing.

Digital light procession (DLP) technology is used to manufacture the soft actuator body. DLP technology is an additive manufacturing technology that solidifies a photosensitive polymer liquid layer-by-layer through a laser. With this molding technology, the model is first cut using slicing software, and the slide is read by the laser. The thin area of the resin layer produces the same photopolymerization reaction as the slide image, forming a thin layer on the part. Then, the molding table moves down one layer, and the laser reads the next slide, finally molding the desired object. The bionic actuator is made of photosensitive resin and uses a 405-wavelength UV stereolithography device. The performance parameters of the resulting photosensitive resin are as follows: hardness: 60–75 HRA; viscosity: 980 mPa.s at 25 ℃; tear strength: 47.2 KN/m; tensile strength: 7.9 MPa; Young’s modulus: 2.0 MPa; and elongation at break: 255.1%.

Step 2: Use a laser to cut a carbon fiber plate of 0.5 mm thickness suitable for the actuator.

Step 3: Glue the actuator body to the carbon fiber plate, as shown in Figure 4c. The use of a carbon fiber plate improves the plane stiffness of the structure. However, the motion of the soft robot will be inaccurate because of the softness of the soft actuator. The use of carbon fiber plate in the design causes the actuator to be rigid and flexible on the whole, with higher motion accuracy, and it can effectively avoid spheroidization. The shape of the carbon fiber plate is the same as the plane, and the distance between each edge of the carbon fiber plate and each edge of the flexible body is 5 mm when installed, which ensures each edge is flexible, easy to fold, and provides the actuator with precise movement.

## 3. Results

### 3.1. Large Torque Output

**Figure 5:**
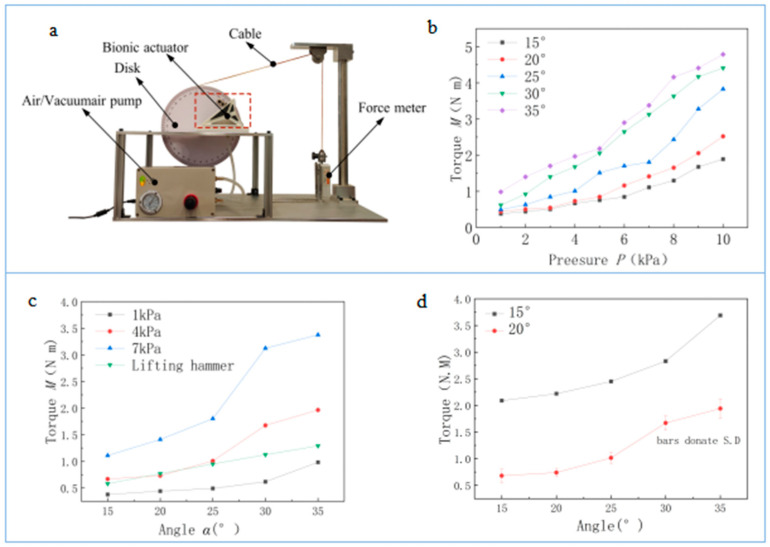
Comparison of theoretical analysis results with experimental results. (**a**) Testing device. (**b**) Measurement of the output torque (*M*) at pressures from 1 to 10 kPa and angles (*α*) from 15° to 35°. The results show that the torque (*M*) increases with increasing pressure, and the torque rate increases with increasing pressure. (**c**) Based on the data analysis, when the pressures are 1, 4, and 7 kPa, the larger the angle (*α*) at the same pressures, the greater the output torque.(**d**) Comparison of theoretical analysis and experimental results.

A bionic actuator was designed and manufactured with a maximum *α* of 40° and a wall thickness of 0.5 mm, L=135 mm, W=80 mm, S=45 mm, a=29 mm, b=85 mm, and h = 8.5 mm. To test the torque of the actuator under different pressures and angles, an experimental test device was set up, as shown in Figure 5a. The experimental instruments include an experimental bench, an actuator, an air pump, a cable, a force meter, and a disk with a dial diameter of 260 mm that could be rotated around the rotational center. The rectangular plane (I) of the bionic actuator was fixed on the experimental bench, and another rectangular plane (I) was connected with a disk connected to a cable. The other end of the rope was connected to a force meter. The actuator was at a different initial angle, the cable was stretched and connected with the force meter, and gas with different pressure values filled the actuator via the air pump. However, because of tension in the cable, the actuator did not produce movement, record the force measured under different pressures and angles, or convert it into torque. The torque (*M*) is a product of the disk radius and the measured force. The above experiment was repeated 10 times, and the average value was taken as the result.

The results show that torque (*M*) increases with increasing pressure (*p*), as per Figure 5b. Under the same pressure, the larger the angle (*α*), the greater the output torque (*M*), as shown in Figure 5c; the force in the nonmoving direction has a force-amplifying effect in the direction of movement, and the amplifying effect is influenced by *α*. 

The bionic actuator has strong adaptability in certain fields. For example, a mechanical arm that lifts heavy objects is shown in Figure 1c. When a conventional actuator is used under such working conditions, the drag torque applied by the heavy hammer increases as the angle increases. To ensure that the heavy hammer rises steadily, the input pressure of the actuator needs to increase, but controlling the speed of the mechanical arm becomes difficult. As the angle (α) increases to almost 90°, a large amount of pressure must be input, testing the actuator’s pressure tolerance. Because the output torque increases as the angle increases, when the bionic actuator is applied to this working state, it can maintain the stable, low air pressure in the actuator while also meeting the driving requirements, reducing the difficulty of control, and protecting the actuator. There are many machines with similar working characteristics, such as legged robots, and bionic actuators have better adaptability in machines with increasing drag torque. By comparing the theoretical analysis data with the experimental results, as shown in Figure 5d, it can be seen that the torque value of the theoretical analysis is larger than the value measured in the experiment. This is because of the higher wall thickness of the actuator during the experiment, and part of the inner surface of the actuator did not experience the effect of gas pressure at a small angle, which led to the lower experimentally measured torque value. At the same time, both the theoretical and experimental data show that as the angle increases, the torque changes.

### 3.2. Bidirectional Drive Performance

**Figure 6:**
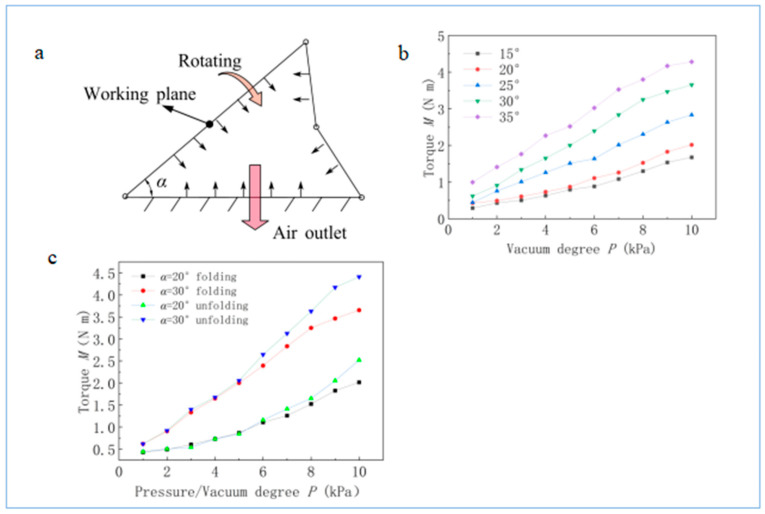
(**a**) Simplified stress diagram. (**b**) Correlation between torque (*M*) and inner vacuum degree (*P*). When folding, *P* represents the pressure difference between the inside and outside, reflecting the pulling force generated by the vacuum. (**c**) The results for torque (*M*) change trends during unfolding and folding, which are similar.

As shown in Figure 6a, we pumped out the internal air using a vacuum air pump and measured the degree of internal vacuum using a vacuum gauge. We measured the torque (*M*) at different angles (*α*) and different vacuum degrees (*p*), as shown in Figure 6b. The results show that when the actuator folds, its torque (*M*) is similar to when the actuator unfolds; however, *M* is slightly smaller when the actuator folds, as shown in Figure 6c. This is because the bionic actuator is fully unfolded in its natural state; furthermore, the minute elastic deformation force in the actuator hinders movement in the direction of folding, though this elastic deformation force assists movement in the direction of unfolding, which is due to the small size of the materials and structures. Therefore, this influence can be eliminated when the size is increased.

### 3.3. Steady-State Analysis

**Figure 7:**
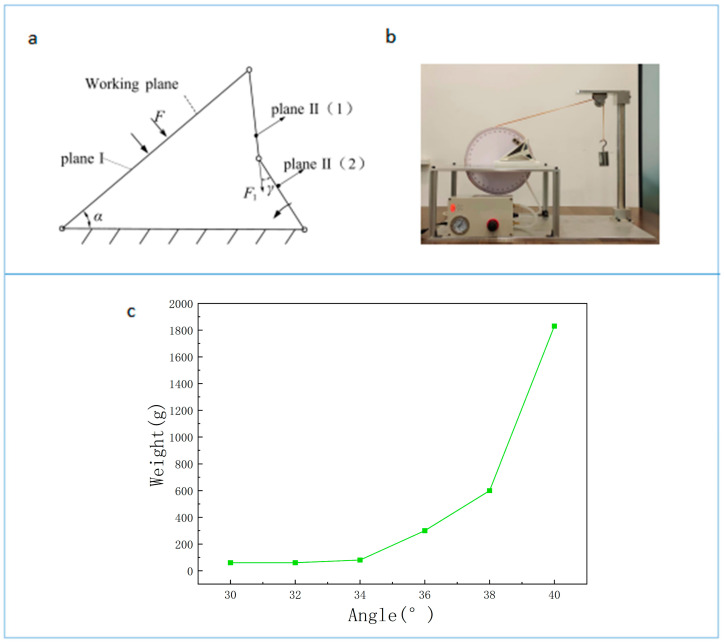
(**a**) Rectangular plane II is divided into plane II (1) and plane II (2) along the crease, and the force (*F*) applied by the load is transmitted through plane II (2) to plane II (1). Force $F_{1}$ is generated in plane II (1), turning it counterclockwise so that the bionic actuator cannot maintain its original shape. The transmission angle (γ) when transmitted from plane II (2) to plane II (1) is shown in the figure. (**b**) Experimental verification. (**c**) Experimental result.

When the internal and external pressures of the bionic actuator are equal, the bionic actuator is in a free state. In general, the actuator is connected to particular structures, and the presence of these structures applies a certain load to the working plane. As shown by *F* in Figure 7a, rectangular plane II is divided into plane II (1) and plane II (2) along the crease. If plane II (1) and plane II (2) are coplanar, the force (*F*) cannot cause the bionic actuator to rotate and reach a steady state. Therefore, when the bionic actuator is fully unfolded or folded, plane II (1) and plane II (2) are coplanar, and the structure is now bistable. When it reaches a steady state, the bionic actuator can withstand the force applied by the load on plane I and maintain a stable structural state without applying additional force to the bionic actuator, which is very useful in many devices that need to maintain structural stability when unfolded or folded. To verify this conclusion, we conducted relevant experiments. The specific experimental steps are as follows: Produce a actuator with the following parameters: L=135 mm, W=80 mm, S=45 mm, and h=10 mm. Plane II (1) and plane II (2) are coplanar in the free state. Install the device, as shown in Figure 7b, and turn off the air supply switch such that the actuator maintains the same internal and external air pressure. Since α is at a maximum of 40°, when the actuator is manufactured, the free state of the actuator is such that when α is at a maximum, the actuator is in a stable state. With this as the initial state, hang a weight on the rope; the weight of the weight starts at 20 g, and 10 g is added each time. The weight of the weight is recorded when the actuator moves, and the initial state of the actuator is then changed. The experiment is repeated at different initial angles, and the data at different initial angles are recorded, as shown in Figure 7c. The experimental results show that when the initial angle is α = 40°, 1820 g of weight is needed to fold the actuator. As the initial angle is reduced, the weight required to fold the actuator decreases rapidly. This phenomenon verifies the correctness of the above theoretical analysis results.

The approximately quasi-triangular prism structure can achieve a bistable state by adjusting the size of the structure in the unfolded state and the folded state [30]. Given the properties of the bionic actuator, such as a small volume, large amount of torque, bidirectional driving ability, and stable structure, it has a broad range of applications. Bionic actuators can be a good solution for devices when they need to be small during transport or storage and large during use. For example, space-deployable structures must be in a folded state and fixed in the vehicle cabin to save space during launches. After being launched, based on design requirements, these structures must be gradually deployed and become large, remaining in this state.

## 4. Discussion

**Figure 8:**
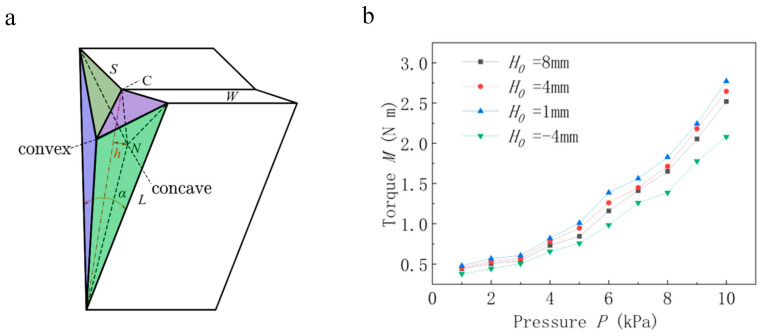
(**a**) The triangular plane may be concave or convex. (**b**) When $H_{0}$ is positive under the same pressure, the smaller $\;H_{0}$, the larger the actuator output torque (*M*).

Through calculations and experiments, we verified that rectangular plane II increases torque when it is concave inward, and we believe that the triangular plane has a similar effect on movement. The distance between point *N* and the triangular plane is *h*; *h* is positive when the triangular plane is concave and negative when the triangular plane is convex, and *h = H*_0_ when the angle (α) is at the maximum, as shown in Figure 8a. The relationship between torque (*M*) and the angle (*α*) is discussed below regarding different *H_0_* values.

For the remaining values, the maximum angle is α = 40°, L=135 mm, W=80 mm, and S=45 mm. As such, different $H_{0}$ values can be used, and we can discuss the relationship between torque (*M*) and input air pressure (*P*) for these various $H_{0}$ values at an angle (α) of 20°. The results show that under the same pressure, when *h* is positive, *H*_0_ will be smaller, and torque (*M*) will be larger. However, when *h* becomes negative, the output torque (*M*) drops sharply. Therefore, the application of pressure on the triangular plane, when it is concave, promotes movement, thus confirming the previous conjecture, as shown in Figure 8b. 

## 5. Conclusions

This paper described the design and analysis of a bionic actuator. By rationally designing its structure, shortcomings of the actuator such as fragility, inaccurate movement, low output torque, and an inability to move in more than one direction were effectively overcome, and high-torque bidirectional movement as high as 4.78 N m was realized. The results show that the air pressure acting on the nonworking plane can promote motion, and torque increases with an increase in the unfolding angle. The results also show that the torque properties of the bionic actuator are similar during unfolding and folding. In addition, the theoretical research shows that the bionic actuator is bistable. When it reaches a stable state, the bionic actuator can withstand the force of the load applied to plane I and maintain its stable structural state without applying any additional force, which is very useful in many devices where maintenance of structural stability is necessary when unfolding or folding. Finally, by setting different positions for point *N*, the influence of the triangular plane on torque characteristics can be determined. The results show that pressure on the triangular plane, when it is concave, promotes movement.

## Data Availability

Not applicable.
